# Percutaneous Dye Image Cardioscopy for Detection of Endocardial Lesions

**DOI:** 10.1155/DTE.7.29

**Published:** 2000

**Authors:** Masahito Kanai, Takeshi Sakurai, Kunio Yoshinaga, Kaneyuki Aoyagi, Takashi Hitsumoto, Masaki Yoshinuma, Takashi Uchi, Hirofumi Noike, Hidefumi Ohsawa, Kouhei Kawamura, Keiichi Tokuhiro, Makiko Takahashi, Tadashi Ebihara, Keiichi Tachihara, Yasumi Uchida

**Affiliations:** 1 Cardiovascular Center 564 Shioshizu Sakura-shi Chiba 285-0841 Japan; 2 Clinical Engineering Toho University Hospital at Sakura 564 Shioshizu Sakura-shi Chiba 285-0841 Japan

## Abstract

Endocardial lesions are caused not only by inflammatory processes but also by myocardial ischemia, resulting in endocardial thrombosis and cerebral embolism. We deviced a method for direct visualization of endocardial damages by a novel dye image cardioscopy with Evans blue and examined its feasibility in patients with heart disease. The dye was injected into the left ventricle before and after endomyocardial biopsy. Endocardial surface was stained in dark blue in 63% of patients with angina pectoris before biopsy. After biopsy, the biopsied portions were stained in blue in all. The results indicate that endocardium is damaged even in apparently intact LV in patients with ischemic heart disease and that endomyocardial biopsy causes severe endocardial damages.


*Diagnostic and Therapeutic Endoscopy*, Vol. 7, pp. 29–33
Reprints available directly from the publisher
Photocopying permitted by license only

© 2000 OPA (Overseas Publishers Association) N.V.
Published by license under
the Harwood Academic Publishers imprint,
part of The Gordon and Breach Publishing Group.
Printed in Malaysia.

### Percutaneous Dye Image Cardioscopy for Detection of Endocardial Lesions

MASAHITO KANAI[a,*], TAKESHI SAKURAI[a], KUNIO YOSHINAGA[a], KANEYUKI AOYAGI[a], TAKASHI HITSUMOTO[a], MASAKI YOSHINUMA[a], TAKASHI UCHI[a], HIROFUMI NOIKE[a], HIDEFUMI OHSAWA[a], KOUHEI KAWAMURA[a], KEIICHI TOKUHIRO[a], MAKIKO TAKAHASHI[b], TADASHI EBIHARA[b], KEIICHI TACHIHARA[b] and YASUMI UCHIDA[a]

[a]*Cardiovascular Center,* [b]*Clinical Engineering Toho University Hospital at Sakura, 564 Shioshizu, Sakura-shi, Chiba 285-0841, Japan*

*(Received 3 May 2000; Revised 15 June 2000; In final form 15 June 2000)*

**Endocardial lesions are caused not only by inflammatory processes but also by myocardial ischemia, resulting in endocardial thrombosis and cerebral embolism. We deviced a method for direct visualization of endocardial damages by a novel dye image cardioscopy with Evans blue and examined its feasibility in patients with heart disease. The dye was injected into the left ventricle before and after endomyocardial biopsy. Endocardial surface was stained in dark blue in 63% of patients with angina pectoris before biopsy. After biopsy, the biopsied portions were stained in blue in all. The results indicate that endocardium is damaged even in apparently intact LV in patients with ischemic heart disease and that endomyocardial biopsy causes severe endocardial damages.**

*Keywords*: Endocardial damage, Evans blue dye, Ischemic heart disease, Percutaneous cardioscopy, Vital staining

### INTRODUCTION

In 1976, Uchida reported a original percutaneous cardioscopic technique, and the clinical application of the cardioscopy was started in 1987. Since then, percutaneous cardioscopy enabled percutaneous observation of the interior of the heart for a macroscopic pathologic diagnosis of the heart disease [1–3]. Clinically, there is a specific syndrome “Cardiocerebral Syndrome” in which cerebral attack occures following cardiac attack (acute myocardial infarction). It is generally believed that thrombus formed in the heart is detached and flows into the cerebral arteries, resulting in this syndrome. However, the process of thrombus formation on the endocardium of the ischemic region of the heart is not well known.

* Corresponding author. Tel.: + 81-43-462-8811, ext. 2215. Fax: + 81-43-487-4246.

Extensive myocardial infarction causes left ventricular contraction disturbance and stagnation of blood. This stagnation is believed to be the main cause of thrombus formation. Recent cardioscopic examination revealed frequent mural thrombus in the left ventricle without obvious contraction disturbance [4]. This fact suggests that blood stagnation is not the sole cause of thrombus formation. There is a possibility that endocardial cells which act against thrombus formation are damaged due to myocardial ischemia resulting in thrombus formation. Therefore, we attempted whether or not endocardial damages exist in patients with ischemic heart disease.

Discrimination of endocardial cell damages are beyond conventional cardioscopes. We found that Evans blue dye which selectively stains endothelial cells of the cardiovascular systems. Therefore, we performed dye image cardioscopy in patients with ischemic heart disease.

### SUBJECTS AND METHODS

### Cardioscope system

The cardioscope system was composed of a thin 5F fiberscope (Olympus Co, LTD, Tokyo, Japan) and a 9F guiding balloon catheter (Fig. 1). The fiberscope was connected to a CCD camera and an illumination source (Xenon lamp). The obtained images were displayed through a video converter simultaneously with X-ray images on a 14-inch video monitor and were recorded on a vide tape.

### Subjects

Thirty-five patients with heart disease (16 pts with angina pectoris (AP), 11 with dilated cardiomyopathy (DCM) and 8 pts with chest pain syndrome (CPS)) underwent percutaneous dye image

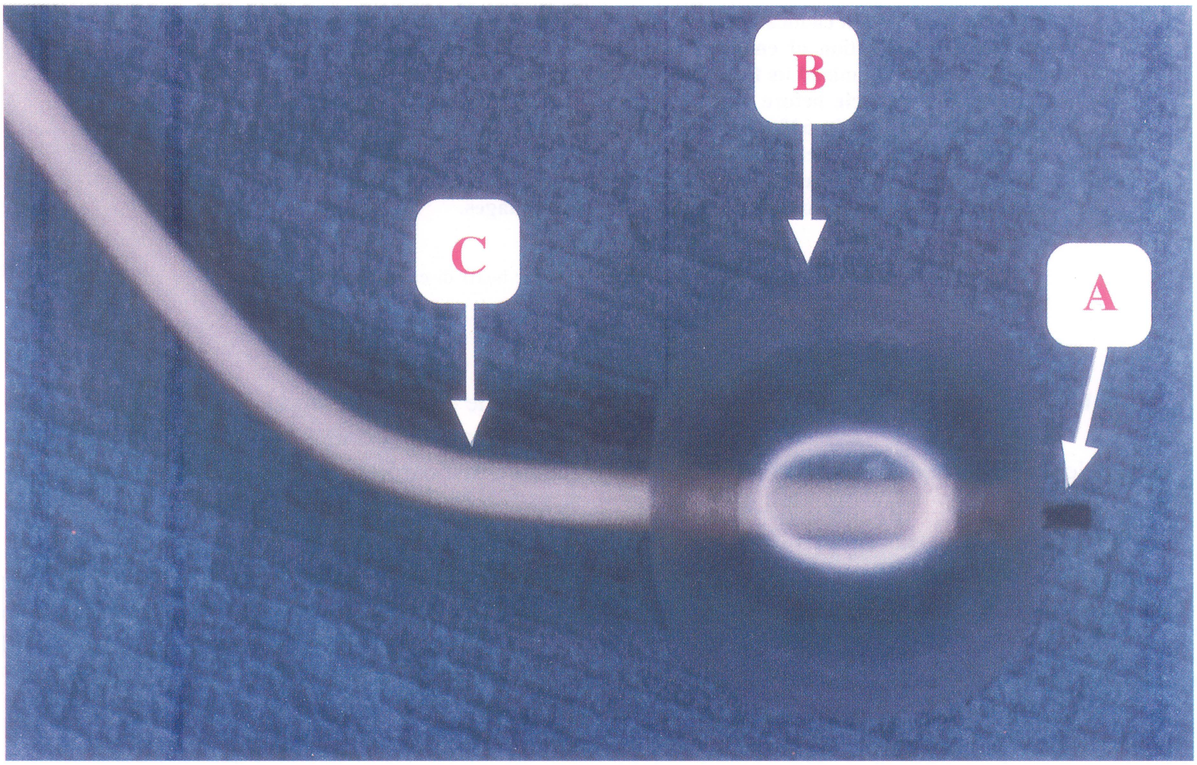

FIGURE 1 Cardioscope used in this study. (A) fiberscope 5F (Olympus Co. Ltd., Tokyo, Japan) (B) balloon (C) shaft.

cardioscopy after the routine cardiac catheterization. Because of the experimental nature, informed consent for percutaneous dye image cardioscopy was obtained from all patients.

### Procedures

After the routine catheterization and angiography, a guiding balloon catheter was introduced, through the sheath previously introduced into the right femoral artery, into the left ventricle. The balloon was inflated with $CO_2$. Then, a 5F fiberscope was introduced through the catheter so as to locate its tip at the distal most tip of catheter. The inflated balloon was pushed against the left ventricular wall. Heparinized saline was infused at a rate of 3–5 ml/sec for several seconds by a power injector for observation. Thereafter, 1 ml of Evans blue dye solution (20 mg/ml saline solution) was directly injected through the guiding catheter into the left ventricle.

### RESULTS

Figure 2 shows the endocardial surface in a patient with angina pectoris. The surface was apparently intact before staining. After staining, however, the surface was stained in dark blue.

Figure 3 shows the edges of the trabeculae in a patient with angina pectoris. The edges were white in color before staining. After staining, the edges were stained in dark blue.

Figure 4 shows the edges of the trabeculae in a patient with dilated cardiomyopathy. The edges of trabeculae were atrophic and yellow in color before biopsy. After biopsy, the biopsied portion was extensively stained in dark blue.

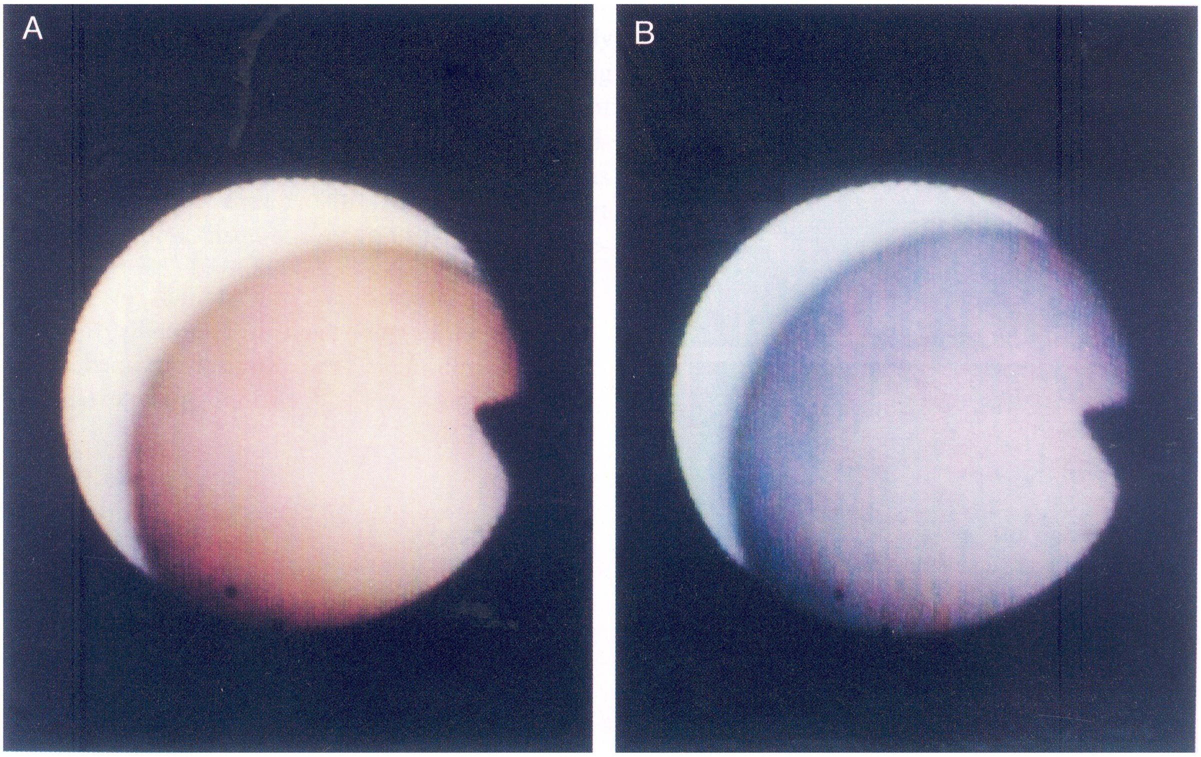

FIGURE 2 47-year-old female with angina pectoris. (A) The endocardial surface was brown in color before staining. (B) The surface was stained in dark blue with Evans blue.

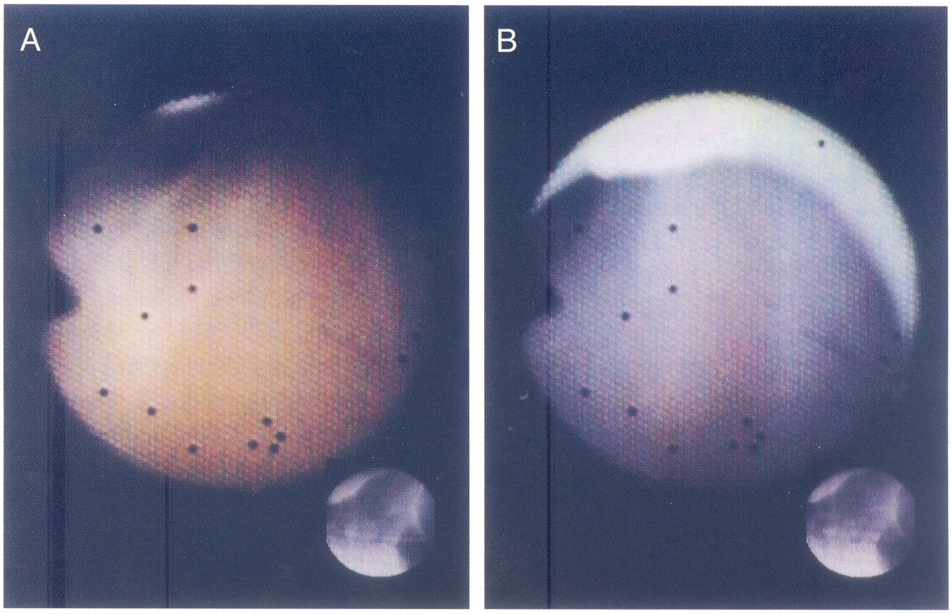

FIGURE 3 69-year-old male with angina pectoris. (A) The edge of trabecle was white in color before staining. (B) The endocardial surface was stained in dark blue with Evans blue.

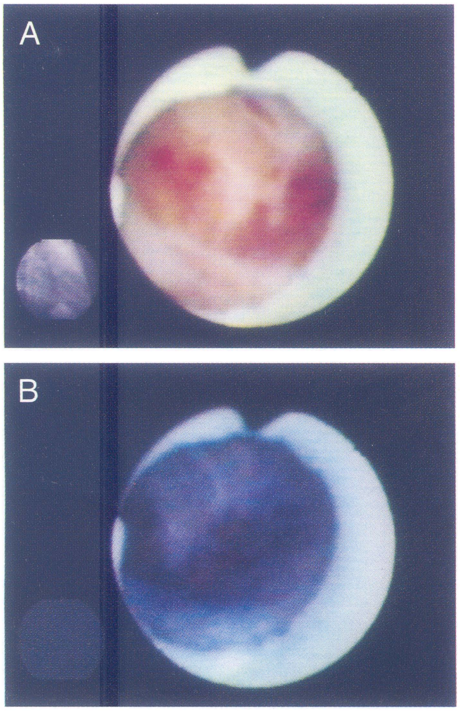

FIGURE 4 44-year-old male with dilated cardiomyopathy. (A) The trabecle (arrow) was atrophic and yellow in color before biopsy. (B) The endocardium and thrombus (arrow) were stained in dark blue after biopsy.

TABLE I Endocardial staining with evans blue dye

| | Before biopsy | After biopsy |
|---|---|---|
| AP | 10/16 (63%) | 4/4 (100%) |
| DCM | 8/11 (73%) | 6/6 (100%) |
| CPS | 3/8 (38%) | 1/1 (100%) |

AP: angina pectoris, DCM: Dilated cardiomyopathy. CPS: Chest pain syndrome.

Thus, endocardium was stained in 63% of patients with angina pectoris. Especially, the edges of the trabeculae were strongly stained. After biopsy, the biopsied portions were extensively stained (Table I).

### DISCUSSION

Recent advances in fiberscope and guiding system technology enabled us to perform a percutaneous observation of the luminal changes of the cardio-vascular systems [5–8]. However, the conventional cardioscope could not allow discrimination of fine composition of the target. It was beyond the ability of conventional cardioscope for differentiation of the cardiovascular changes of cellular order. Therefore, to observe vascular luminal changes of cellular order, an intravascular microscope was devised, and its feasibility was examined. The luminal changes observed by the microscope correlated well with histologic changes [9–10]. Furthermore, structures of human vascular lesions of cellular order could be observed percutaneously by the intravascular microscope [11].

However, the microscope is composed of a rigid rod lens but not of flexible thin glass fibers, and its length is short. Therefore application to the heart is impossible. In this study, in order to discriminate endocardial changes of cellular order, we devised percutaneous dye imaged cardioscopy. With the aid of Evans blue, endocardial damages were clearly demonstrated.

In this study, endocardial damages were frequently observed in patients with angina pectoris as in case of DCM. Inflammatory process is considered to be involved in DCM. In case of angina pectoris, severe ischemia may affected not only myocardial cells but also endocardial cells, leading to their death and fibrosis. These otherwise not demonstrable endocardial damages may contribute to mural thrombus formation, which was not infrequently observed in this category of heart disease. These thrombus may participate in cause-unknown cerebral thrombo-embolic attacks.

### *References*

[1] Uchida, Y., Fujinori, Y. and Hirose, J. Percutaneous left ventricular endomyocardial biopsy with angioscopy guidance in dilated cardiomyopathy. *Am. Heart J.* 1989; **118**: 1039–1041.
[2] Uchida, Y., Tomaru, T., Nakamura, F. *et al.* Percutaneous fiberoptic cardioscopy of the left ventricle in patients with ideopatic myocarditis and dilated cardiomyopathy. *Am. Heart J.* 1990; **119**: 949–950.
[3] Oshima, T., Hirose, J., Sasaki, T. and Uchida, Y. Cardioscopic observation of cardiac thrombus. *Circulation* 1994; Supple: **50**.
[4] Uchida, Y. Mural thrombus in the heart. *Cardioangioscopy* 1995; 126–129.
[5] Uchida, Y., Tomaru, T., Nakamura, F. *et al.* Percutaneous coronary angioscopy in patients with ischemic heart disease. *Am. Heart J.* 1987; **114**: 1069–1075.
[6] Sherman, C.T., Litvak, F., Grundfest, W. *et al.* Coronary angioscopy in patients with unstable angina pectoris. *N. Engl. J. Med.* 1989; **60**: 913–919.
[7] Ramee, S.R., White, J.C., Collins, T.J. *et al.* Percutaneous angioscopy during angioplasty using a steerable microangioscopy. *J. Am. Coll. Cardio.* 1991; **17**: 100–105.
[8] Uchida, Y., Fujinori, Y., Tomaru, T. *et al.* Percutaneous angioplasty of chronic obstruction of peripheral arteries by a temperature-controlled Nd:YAG laser system. *J. Intervent Cardiol.* 1992; **5**: 301–308.
[9] Uchida, Y., Tomaru, T., Kamijo, T. *et al.* Comparative studies on transluminal angiomicroscopic and ex vivo microscopic features of vascular lesions. *SPIE* 1992; **1642**: 217–220.
[10] Tomaru, T., Uchida, Y., Nakamura, F. *et al.* Transluminal angio-microscopy of arterial thrombus and thrombolysis. *SPIE* 1992; **1642**: 214–216.
[11] Uchida, Y., Nakamura, F. and Morita, T. Observation of atherosclerotic lesions by an intravascular microscope in patients with arteriosclerosis obliterans. *Am. Heart J.* 1995; **130**: 1114–1119.

